# The effects of functional fiber on postprandial glycemia, energy intake, satiety, palatability and gastrointestinal wellbeing: a randomized crossover trial

**DOI:** 10.1186/1475-2891-13-76

**Published:** 2014-07-28

**Authors:** Jannie Yi Fang Yuan, Rebecca Jane Mason Smeele, Kate Daisy Harington, Fiona Maria van Loon, Anne Jacoba Wanders, Bernard Joseph Venn

**Affiliations:** 1 Department of Human Nutrition, University of Otago, PO Box 56, Dunedin 9054, New Zealand; 2 Wageningen University, Wageningen, Netherlands

**Keywords:** Fiber, Functional, Bread, Satiety, Glycemia

## Abstract

**Background:**

Fiber intakes in developed countries are generally below those recommended by relevant authorities. Given that many people consume fiber-depleted refined-grain products, adding functional fiber will help to increase fiber intakes. The objective of the study was to determine metabolic and sensory effects of adding fiber to bread.

**Methods:**

A double-blind pair of randomized crossover trials with a two-week washout in which two fiber-containing breads were compared with control bread. The functional fiber (fruit fiber and FibreMax™) was added to yield 10 g fiber per serve (two slices). Eighty participants (n = 37 fruit fiber and n = 43 FibreMax™) consumed one serve of bread (fiber or control) followed three hours later by a pasta meal consumed *ad libitum*. Outcome measures included glycemia, satiety, palatability, gastrointestinal wellbeing, visual appeal and subsequent energy intake of the pasta meal. Multivariate regression was undertaken to test for differences between treatment and control for blood glucose, satiety, and cumulative energy intake. Satiety responses were also compared by splitting the data into an immediate response after eating (0–30 min) and a return to hunger analysis (30–180 min). A Wilcoxon sign rank test was used for the first component (0–30 min) and Wilcoxon matched-pairs signed-rank test for the second component (30–180 min). Between treatment differences for gastrointestinal wellbeing were tested using Pearson’s chi-square test or Fisher’s exact test.

**Results:**

Consumption of the fruit fiber bread reduced postprandial glycemia by 35% (95% CI 13 to 51; P = 0.004) and cumulative energy intake by 368 kJ (95% CI 163 to 531; P = 0.001). There was little influence on satiety and the bread was rated as having poor taste and smell whilst generating feelings of nausea in some participants. FibreMax™ enriched bread reduced glycemia by 43% (95% CI 17 to 61; P = 0.004) without influence on energy intake or satiety. Apart from a lower visual appeal, the FibreMax™ bread was palatable. Neither bread caused gastrointestinal discomfort related to flatulence or bloating.

**Conclusions:**

Enriching bread with 10 g of functional fiber per serve is feasible although reformulation is needed to create not only an acceptable bread, but a desirable product.

## Background

Diabetes and cardiovascular disease were among the top 10 leading causes of death worldwide between the years 2000 and 2011 [1]. There is evidence that fiber can improve risk factors for these chronic conditions including having positive effects on blood glucose concentrations and body mass [2,3]. Consuming long-term intakes of up to 15 g/day of either natural or functional fibers is associated with significant reductions in fasting glycemia [2]. Additionally, a high fiber diet has the potential to reduce body mass through its ability to improve satiety and decrease energy intake [3]. From previous work, 14 g/day of either natural or functional fibers was associated with a 10% decrease in energy intake and a 1.9 kg reduction in body mass over 4 months [3].

In light of the potential health benefits, populations have been encouraged to consume more fiber. However, current fiber intakes have been low with some populations in developed countries consuming as little as 40% of recommended target intakes with New Zealanders consuming around 70% of the target [4,5]. This may reflect the difficulty in increasing fiber intake through natural food alone due to barriers such as having a busy lifestyle and a lack of time to prepare healthier food, or taste preferences for refined carbohydrates [6]. Additionally, fiber has been associated with bloating, flatulence, and abdominal rumbling [7], which can further discourage its intake. Given this shortfall in fiber intake and possible reluctance to change eating habits, food fortified with functional fiber that is largely undetectable to the consumer in terms of taste and texture could provide a good strategy for increasing fiber intakes.

In recent years, manufacturers have been developing and selling various fiber products. However, inconsistent results have been found for the relationship between fiber and glycemia [8], and between fiber, energy intake, satiety, and body weight [9]. Additionally, much of the research has been focused on soluble fiber with less data available on insoluble or blends of soluble/insoluble mixed fibers. Given the availability of insoluble or mixed fiber products currently on the market, which at times is accompanied by statements suggesting glycemic and weight loss benefits [10], more research on the effects of these fibers on blood glucose response and satiety is warranted. Therefore, the objective of this study was to determine metabolic and sensory effects resulting from the enrichment of a serving of bread with an amount of functional fiber sufficient to raise the average consumer’s fiber intake to recommended levels, with an ultimate goal of providing a better bread option to people who currently choose fiber-depleted bread.

## Methods

### Subjects

A total of 83 university students aged between 18–35 years (mean 21 years; SD 2.8), with normal body mass index (mean 22.5 kg/m^2^; SD 2.7), and normal fasting blood glucose (mean 4.6 mmol/l; SD 0.5) were recruited. Exclusion criteria included being diagnosed with chronic or digestive diseases, food allergies, pregnancy, and taking medications or supplements likely to influence glucose metabolism or gastrointestinal wellbeing. The study was approved by and conducted in accordance with the ethical standards set by the University of Otago Human Ethics Committee.

### Study design

The experiment consisted of two randomized double-blinded crossover trials of two fiber breads: 1). A prototype product derived from fruit supplied by Anagenix (Petone, New Zealand) and 2). FibreMax™ (New Image International, Auckland, New Zealand). The fruit fiber was predominately an insoluble, non-viscous fiber. FibreMax™ contained a mix of soluble and insoluble polysaccharides that formed a gel with water comprising chicory root extract, psyllium, soy fiber, oat bran and pectin. A quantity of 15 g of powder for both products provided approximately 10 g of fiber. Participants were randomly assigned to consume a control bread, and either a fruit fiber or FibreMax™ bread during separate sessions separated by a two week washout. The serving was two slices of bread with 10 g of margarine (Craig’s, Heinz Wattie’s Ltd) accompanied by 250 ml of water. Bread and water were consumed within 15 minutes. A standard protocol for fasting and a recommendation to include carbohydrate in the meals prior to test days was given. Additionally, participants were reminded to avoid alcohol and vigorous physical activity prior to clinic days.

### Test bread

Each loaf of bread was prepared with the following ingredients: white wheat flour (604 g or 820 g; control or fiber-enriched bread), added fiber (0 g or 216 g; control or fiber-enriched bread), 15 g salt, 56 g oil, 60 g sugar, 14 g yeast together with 400 mL of water in the control bread and 1000 ml or 700 ml water in the fruit fiber and FibreMax™ bread, respectively. Bread was baked in batches, sliced and frozen. On test days, the bread was defrosted at room temperature for at least five minutes before use. Nutrient compositions of the bread are summarized in Table 1. Gribbles Veterinary (Dunedin, NZ) conducted chemical analyses using AOAC and Pearson chemical analysis methods [11,12].

**Table 1:** Nutrient composition of treatment and control bread per serving size

	**Fruit fiber**	**Control**	**FibreMax™**	**Control**
Weight per serving (g)	133 (128, 138)	105 (100, 110)	123 (118, 128)	92 (87, 97)
Energy (kJ)	1000	1267	1338	1113
Protein (g)	8.8 (14.9)	10.9 (14.7)	9.3 (11.9)	9.6 (14.6)
Fat (g)	0.9 (3.4)	5.8 (16.9)	5.0 (13.9)	4.5 (15.0)
Available CHO (g)*	34.2 (58.1)	47.6 (63.8)	45.0 (57.2)	42.6 (65.1)
Dietary fiber (g)	13.8 (11.1)	3.5 (2.2)	13.4 (8.0)	3.5 (2.5)
Soluble (g)	2.1 (1.7)	0.3 (0.2)	4.3 (2.6)	0.3 (0.2)
Insoluble (g)	11.7 (9.4)	3.2 (2.0)	9.1 (5.4)	3.2 (2.3)
Moisture (ml)	73.5	35.7	48.0	30.5

*Avail CHO = total carbohydrate – dietary fiber.

### Postprandial glycemia

Testing protocols were conducted in accordance to recommendations by glycemic methodology guidelines and papers [13]. A capillary blood sample was collected at baseline (immediately before bread consumption) and thereafter at 30, 45, 60, 90, and 120 minutes. A calibrated glucose analyzer (HemoCue® Glucose 201+, HemoCue®; Sweden) was used to measure glucose concentrations.

### Subsequent pasta meal and energy intake

An *ad-libitum* pasta meal together with unrestricted water was offered to participants three hours after bread consumption. The pasta meal was prepared the day before each clinic session, stored in the fridge, and reheated in a covered container for 120 minutes at 100°C. Each pasta meal consisted of 550 g of cooked pasta (Pennette, Colavita™) mixed with 230 g of pasta sauce (Buitoni Rich tomato sauce, Nestle™). Pasta was prepared by boiling two parts pasta and three parts water for 10 minutes, rinsed under cold water and then left to stand for 15 minutes. Every 100 g of pasta meal included: 22.2 g carbohydrate, 4.0 g protein, 2.4 g fat, and 535.9 kJ energy. Participants were instructed to eat until they were comfortably full. It was permitted to take leftovers home to minimize temptations to over-consume free food. Meals were weighed to the nearest gram before and after consumption to determine total pasta intake.

### Satiety

Visual Analogue Scales (VAS) previously validated for use in postprandial single meal studies [14] were adapted and used to assess satiety and thirst. The VAS consisted of nine scales 100 mm long that were used to access participants’ level of hunger (0 = not hungry, 100 = very hungry), satisfaction (0 = satisfied, 100 = unsatisfied), fullness (0 = full, 100 = not full), prospective food intake (0 = nothing, 100 = a lot), desire for various food flavors: sweet, savory, salty, or fatty food (0 = no desire, 100 = high desire), and thirst (0 = not thirsty, 100 = thirsty). VAS for satiety were completed at 0 (immediately before bread consumption) and thereafter at 30, 60, 90, 120, 150, and 180 minutes. Additionally, since fiber may interact with water and influence thirst, this parameter was also measured.

### Palatability and gastrointestinal wellbeing

Palatability was assessed using VAS 100 mm in length (0 = dislike, 100 = like). These scales were adapted from one used in a previous study [14] to include texture, as this was relevant for evaluating the fiber-enriched bread. Each participant completed a set of scales immediately following bread consumption to assess visual appeal, smell, taste, texture, aftertaste (0 = none, 100 = a lot), and overall pleasantness of the bread.

Gastrointestinal discomfort was evaluated using questionnaires completed at 0 (immediately before bread consumption) and thereafter at 60, 120, 180 minutes, 8 and 24 hours. Questions were adapted from a validated assessment tool [15] and modified to include symptoms that may be experienced with high fiber intakes [16]. Factors evaluated were the occurrence (‘none’, ‘mild’, ‘moderate’, or ‘quite a lot’) and severity (‘severe’, ‘very severe’, or ‘unbearable’) of any bloating, abdominal rumbling, flatulence, abdominal pain, nausea, and vomiting; stool frequency and consistency were reported over 24 h in accordance with the Bristol Stool Scale [17].

### Statistics

STATA (Stata Statistical Analysis Software, version 10.1, Stata Corporation 2008, USA) was used for statistical analyses and Microsoft Excel (Microsoft Excel, version 14.3.2, Microsoft Corporation 2010, US) to calculate AUC (area under the curve) and IAUC (incremental area under the curve). The primary endpoint for glycemia was change in postprandial glucose IAUC, measured using the trapezoidal method. A multivariate general least square regression for random effects was undertaken on log-transformed IAUC to detect differences between treatment and control; log-transformation is recommended when the variance is dependent on the mean [18]. Differences in estimated incremental peak glucose was also measured as a secondary outcome, and analyzed using a paired t-test. A sample size of 31 participants per treatment group provided 80% power to detect a 20% difference in glucose IAUC using a two sided α of 0.05. This sample size was sufficient for measures of energy intake and satiety, palatability and gastrointestinal wellbeing.

For the pasta meals, a t-test was used to detect differences in energy intake from the subsequent meal between fiber and control breads. The effects of treatment on cumulative energy intake was analyzed using a random effects generalized least squares regression. Regression coefficients and 95% confidence intervals were calculated.

Satiety VAS data were analyzed by plotting the VAS scale (mm) on the y-axis and time (minutes) on the x-axis, and determining the AUC using the trapezoidal rule. Responses to perceptions of hunger, satisfaction, fullness and prospective food intake showed high internal consistency (Cronbach’s alpha 0.87-0.97), indicative that VAS results were highly correlated with good internal reliability. Therefore combining the separate VAS components was likely to give a more robust measure such that hunger, satisfaction, fullness, and prospective food intake were grouped to produce an additional measure termed ‘appetite’.

**Figure 1:**
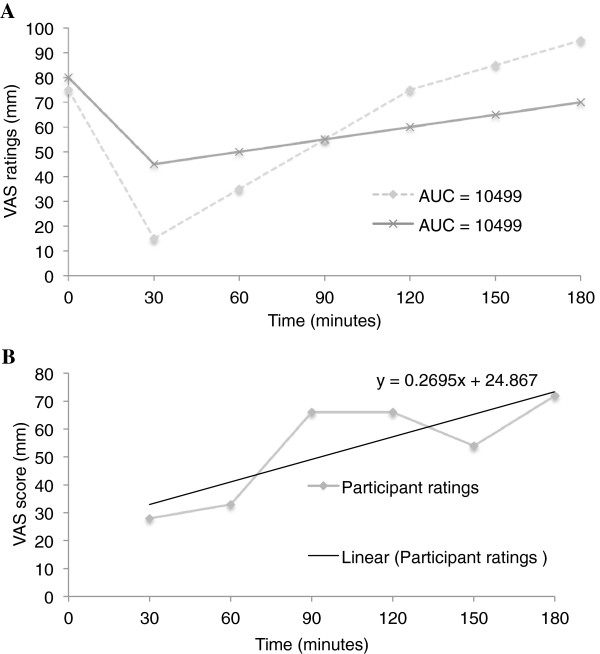
**Insensitivity of area-under-the-curve to differing visual analogue scale ratings. (A)** Example of area-under-the-curve (AUC) insensitivity to visual analog scale (VAS) responses, showing two different shapes with the same AUC; **(B)** Example of VAS return-to-hunger slope calculation 0.2695 mm × (150/2.5) hr = 16.17 mm/hr.

Differences in satiety between treatment and control groups were analyzed using multiple linear regression on logged values. However, although AUC has been recommended as a robust measure for satiety, this analysis can be insensitive to the shape of the response curve [19] with an example depicted in Figure 1. Therefore each set of VAS data was additionally analyzed by being split into two components: [1] change in VAS score (mm) between commencement of eating and 30 minutes later, representing the acute change in appetite sensation after bread consumption, and [2] the line of best fit for satiety data between 30–180 minutes, representing return-to-hunger in mm per hour (Figure 1). Comparisons between treatment and control groups were analyzed using Wilcoxon sign rank tests (for the first component, between 0–30 minutes), and Wilcoxon matched-pairs signed-rank tests (for the second component, between 30–180 minutes).

A Wilcoxon two-sided sign test was used to detect differences in palatability between treatment and control groups. Gastrointestinal wellbeing data was dichotomized into a score indicating acceptable (coded 0, representing ‘none’ or ‘mild’ symptoms) or unacceptable (coded 1, representing ‘moderate’, ‘quite a lot’, ‘severe’, ‘very severe’, and ‘unbearable’) and analyzed using the Pearson’s chi-square test or Fisher’s exact test to determine if there were significant differences between bread types. Logistic regression was used to determine the odds ratio of experiencing gastrointestinal symptoms with fiber compared to control breads. All regression models were adjusted for order of bread consumption. Statistical significance was set to P < 0.05 for a two-sided test unless otherwise stated.

## Results

**Figure 2:**
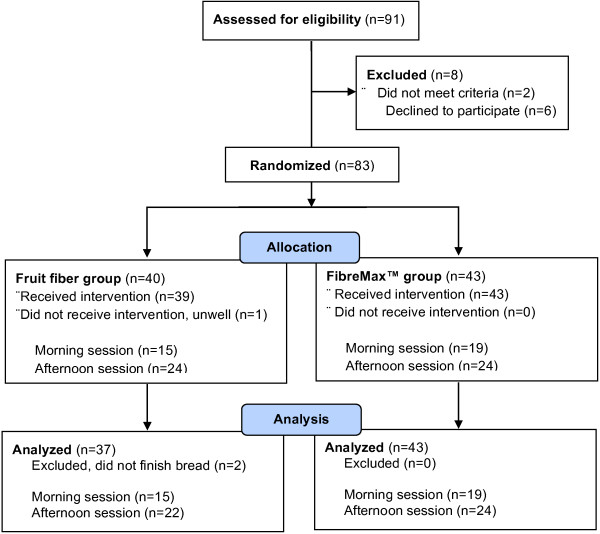
Participant recruitment flow diagram.

A total of 83 participants were randomized with 80 completing the study. One individual dropped out due to feeling unwell, and two others were excluded for finishing less than three quarters of their allocated bread (Figure 2).

### Glycemia

**Figure 3:**
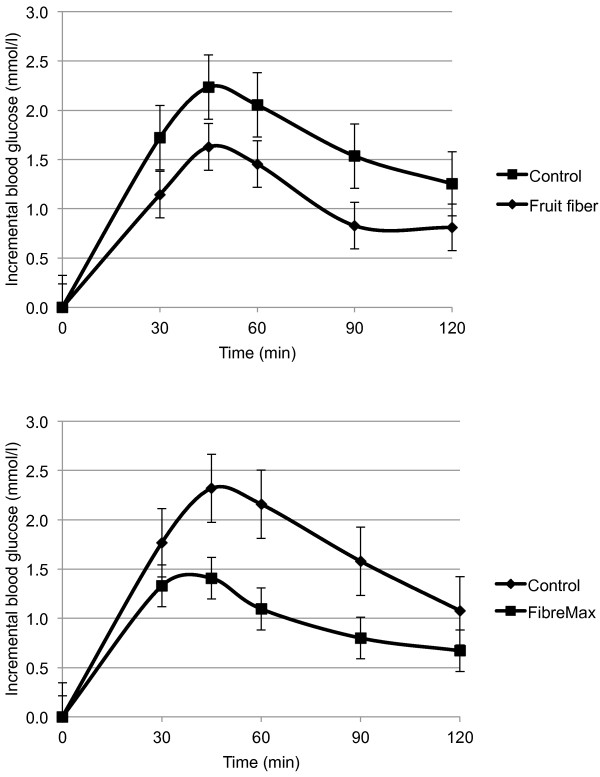
**Postprandial glycemia for fruit fiber group (n = 37) and FibreMax™ group (n = 43).** Data are presented as means with standard error bars.

Postprandial iAUC was 34.9% (95% CI 12.8, 51.3; P = 0.004) less; and 43.1% (95% CI 17.0, 61.1; P = 0.004) less following the fruit fiber and FibreMax™ breads, respectively, compared with control bread. The incremental peak glucose for fruit fiber and FibreMax™ bread was 0.5 mmol/l (95% CI 0.2 to 0.9; P = 0.004) and 0.9 mmol/l (95% CI 0.5, 1.3; P ≤ 0.001) lower than their controls, respectively (Figure 3).

### Subsequent pasta meal and energy intake

There was no difference in baseline appetite scores between treatments; nor was there a difference in pasta or water intake between test days (P > 0.05). The cumulative energy intake (bread plus pasta) was less by 368 kJ (95% CI 163, 531; P = 0.001) on the fruit fiber test day compared with the control test day; there was no difference in cumulative energy intake between the FibreMax™ bread and its control day.

### Satiety

There were no differences in ratings of hunger, satisfaction, fullness, prospective food intake, or appetite between the treatment and control breads. A 20% (95% CI 2, 41; P = 0.029) greater desire to eat fatty food was recorded after eating the fruit fiber bread compared with its control.

### Acute appetite sensation and return-to-hunger slopes analysis

The ratio of the appetite AUC (fiber: control) for FibreMax™ bread was 0.97 (95% CI: 0.88, 1.07); and for fruit fiber bread 0.92 (0.76, 1.10); indicating no difference in this parameter between the fiber breads and their respective control breads. When analyzing the satiety responses over the first 30 minutes after eating, there was no difference in any parameter (hunger, satisfaction, fullness, prospective food intake or appetite) between the FibreMax™ bread and its control. For the fruit fiber bread, there was a decrease in appetite rating from baseline to 30 minutes of 39 (24.5) mm compared with its control bread of 30 (22.9) mm, P = 0.023. The rates of return to hunger (mm/h) over the 30 to 180 minute period following bread consumption were not different when comparing the fiber breads with their respective controls.

### Palatability

The fruit fiber bread had lower scores for smell, taste and overall palatability compared to its control (P < 0.01). Ninety percent of participants rated the aftertaste of the fruit fiber bread as less pleasant than its control bread (P ≤ 0.0001). Only the visual appeal of the FibreMax™ bread scored lower than its control (P = 0.0039).

### Gastrointestinal symptoms

There were no differences in symptoms of bloating, abdominal rumbling, flatulence, abdominal pain, vomiting, or Bristol stool scale between treatments, although the fruit fiber bread was associated with more nausea (occurrence n = 6) than its control (n = 0) (P = 0.02). There were no differences in gastrointestinal symptoms between the FibreMax™ bread and its control.

A summary of metabolic and sensory comparisons between each of the fiber breads and its control bread are given in Table 2.

**Table 2:** Summary of physiological and sensory data comparing the fiber breads with their respective control breads

**Parameter**	**Fruit fiber**	**FibreMax™**
Energy per serve	Lower	Lower
Glycemia	Lower	Lower
Combined energy intake (bread plus pasta)	Lower	No different
Desire to eat salty, sweet, savory foods after bread	No different	No different
Desire to eat fatty foods after bread	Greater	No different
Satiety AUC	No different	No different
Immediate satiety (0–30 min)	Greater	No different
Return to hunger (30–180 min)	No different	No different
Smell, taste, overall palatability	Lower	No different
Visual appeal	No different	Lower
Gastrointestinal effects	No different	No different

## Discussion

The consumption of either the fruit fiber or the FibreMax™ bread reduced postprandial glycemia compared with control bread. In addition, the cumulative energy intake of the fruit fiber bread and a subsequent meal was less compared with its control. Reductions in postprandial glycemia have been found for some soluble fibers, with the effect being attributed to the high viscosity imparted to the food by the added fibers resulting in a reduced rate of gastric emptying and from the entrapment of nutrients in the food matrix [20]. FibreMax™ forms a gel with water, plausibly explaining its glycemic lowering effect. The fruit fiber on the other hand did not gel or form a viscous solution. Glycemic lowering for non-gelling and non-viscous fiber is mostly evident for structurally intact whole grains due to the cereal fiber enclosing the endosperm [21]. In contrast, the mechanism by which the finely ground fruit fiber affected glycemia is most likely a consequence of the displacement of flour with fiber and water (Table 1).

Moderating postprandial glycemia is a desirable outcome with the European Diabetes Policy Group advising that postprandial peak glucose fall within 4.0 - 7.5 mmol/l for healthy individuals and not exceed 9 mmol/l for individuals with diabetes [22,23]. Therefore, even a modest reduction between 0.5 - 1.0 mmol/l in postprandial glucose could be regarded as beneficial to help individuals maintain glucose concentrations within upper recommended limits. Additionally, a physiological rebound of postprandial glycemia below normal background concentrations has been reported with fiber-depleted foods, indicative of poor homeostatic control [24]. A more gradually changing glycemic profile, as evidenced with the fiber containing bread, is metabolically favored [25].

Another parameter we had hoped to affect was satiety. Given that the fiber breads were less energy dense than the control bread, we could have increased the volume of the fiber breads as a means to influence satiety. Our decision to match the breads on serving size rather than on available carbohydrate or weight was because the present study was designed to evaluate the effect of exchanging a serving of fiber-depleted bread with a serving of fiber-enriched bread. From a practical perspective, individuals are more likely to replace one serving of bread with another, rather than exchanging bread based on carbohydrate content or weight. Under our experimental conditions there was no indication for either fiber bread that immediate feelings of hunger substantially differed compared with the control bread. However, a lack of difference in satiety could be regarded as a positive finding given that both fiber-containing breads contained less energy than the respective control breads per serve. The result is particularly encouraging for the fruit fiber bread as the cumulative energy intake of the bread meal combined with a subsequent pasta meal was some 10% less compared with its control. An energy reduction of this magnitude, whilst maintaining satiety, could be useful in an obesogenic environment. To confirm this, further research is warranted to study whether the effects we found after a single exposure leads to sustained satiety and a reduced energy intake after repeated exposures [26].

Our aim was to add around 10 g of fiber to a serving size of bread (5 g per slice) as there is a deficit between the actual fiber intake of New Zealanders compared with that recommended [27]. The addition of either fiber in this amount produced workable dough and loaves that were moist and dense, rising less than the control breads. Adding finely ground fiber maintained the smooth mouth feel of the bread. Nevertheless, there are reservations. The fruit fiber bread had an undesirable aftertaste and slightly increased feelings of nausea, possibly contributing to the participants consuming less of the pasta meal. Formulating and testing a fruit fiber that did not adversely affect palatability and gastrointestinal wellbeing would be essential both to confirm the findings and from a consumer acceptability perspective. Participants rated the palatability of the FibreMax™ bread as acceptable with just its off white color affecting visual appeal. It would be necessary to improve all of these sensory properties given that the purpose of adding fiber is to give a better bread option to people who choose to eat white bread made with fiber-depleted flour.

Despite some sensory shortfalls requiring reformulation, the principle of adding functional fiber appeared to be a feasible option. However, there is debate as to whether the physiological effect of fiber intrinsic to food differs from that of refined and purified fiber [28]. Health authorities around the world recommend consumption of whole grain foods [29] which provide not only more fiber than refined counterparts, but tend to be more nutrient rich overall. Early data supporting the benefits of fiber were taken from studies conducted before the refinement and addition of fiber [30] and there are no equivalent population wide observations in which added or supplemental fiber has been associated with chronic disease. Practically however, there are barriers to consumers choosing whole grain foods including price and taste/texture preference [31]. It is also notable that few products, including whole grain, would contain 10 g of fiber per serve. For consumers of refined foods unwilling or unable to eat whole grain products, the addition of functional fiber to refined bread may provide a better option than a fiber-depleted product. In support of this concept there are good indicators that functional fiber has the potential to influence risk factors for chronic disease. In addition to promoting stool regularity and consistency, food regulatory authorities define functional fiber on the basis of providing acute metabolic effects [32]. Hence, even in the absence of evidence linking added fiber to the prevention of chronic disease, it could be argued that effects on metabolic markers or risk factors is a sufficient basis on which to recommend that added fiber should at least count towards achieving recommended daily fiber intakes.

## Conclusions

Bread was a suitable vehicle for carrying two different functional fibers in an amount that could make a significant contribution to recommended daily fiber intakes. Postprandial glycemia was lessened both by fruit fiber and by FibreMax™ enriched bread compared with control bread. More water and less flour were required in the recipe of the fiber breads compared with the control, resulting in a less energy dense bread on a per serve basis. Effects on satiety and subsequent energy intake were modest. There was a reduction in combined energy intake from the fruit fiber bread and a subsequent pasta meal although this may have been affected by poor palatability. For the fiber breads to be taken up by the consumer, further product development would be necessary to produce bread with not only acceptable, but desirable properties.

## Abbreviations

VAS: Visual analogue scales; AUC: Area under the curve; iAUC: Incremental area under the curve.

## Competing interests

None of the authors have a conflict of interest to declare.

## Authors’ contributions

The authors’ responsibilities were as follows – JYFY, RJMS, KDH, FMVL, AW, and BV designed the research; JYFY, RJMS, KDH, and FMVL conducted the research; JYFY analyzed glycemic data, RJMS analyzed subsequent energy intake data, KDH analyzed satiety data, and FMVL analyzed palatability and gastrointestinal wellbeing data; JYFY wrote the draft manuscript; JYFY and BV had primary responsibility for the final content. All authors read and approved the final manuscript.
